# Comprehensive analysis of 1,771 transcriptomes from 7 tissues enhance genetic and biological interpretations of maize complex traits

**DOI:** 10.1093/g3journal/jkaf140

**Published:** 2025-07-22

**Authors:** Mengyu Lei, Huan Si, Mingjia Zhu, Yu Han, Wei Liu, Yifei Dai, Yan Ji, Zhengwen Liu, Fan Hao, Ran Hao, Jiarui Zhao, Guoyou Ye, Yanjun Zan

**Affiliations:** Tobacco Research Institute, Chinese Academy of Agricultural Sciences, Qingdao, CN 266000, China; Agricultural Genomics Institute at Shenzhen, Chinese Academy of Agricultural Sciences, Shenzhen 518120, China; Tobacco Research Institute, Chinese Academy of Agricultural Sciences, Qingdao, CN 266000, China; College of Ecology, Lanzhou University, Lanzhou 730000, China; Key Laboratory for Bio-Resource and Eco-Environment of Ministry of Education and Sichuan Zoige Alpine Wetland Ecosystem National Observation and Research Station, College of Life Science, Sichuan University, Chengdu, CN 610065, China; Key Laboratory for Bio-Resource and Eco-Environment of Ministry of Education and Sichuan Zoige Alpine Wetland Ecosystem National Observation and Research Station, College of Life Science, Sichuan University, Chengdu, CN 610065, China; Biostatistics Department, School of Public Health, University of Michigan, Ann Arbor, MI 48105, USA; Tobacco Research Institute, Chinese Academy of Agricultural Sciences, Qingdao, CN 266000, China; Tobacco Research Institute, Chinese Academy of Agricultural Sciences, Qingdao, CN 266000, China; College of Forestry, Northwest Agriculture and Forestry University, Yangling, CN 712100, China; College of Information Engineering, Northwest Agriculture and Forestry University, Yangling, CN 712100, China; Tobacco Research Institute, Chinese Academy of Agricultural Sciences, Qingdao, CN 266000, China; CAAS-IRRI Joint Laboratory for Genomics-Assisted Germplasm Enhancement, Agricultural Genomics Institute in Shenzhen, Chinese Academy of Agricultural Sciences, Shenzhen 518120, China; Tobacco Research Institute, Chinese Academy of Agricultural Sciences, Qingdao, CN 266000, China; Department of Plant Physiology, Umeå Plant Science Centre and Integrated Science Lab, Umeå University, Umeå 901 87, Sweden

**Keywords:** GWAS, regulatory variants, integrative genomics, complex trait

## Abstract

By reanalyzing 1,771 RNA-seq datasets from 7 tissues in a maize diversity panel, we explored the landscape of multi-tissue transcriptome variation, evolution patterns of tissue-specific genes, and built a comprehensive multi-tissue gene regulation atlas to understand the genetic regulation of maize complex traits. Through an integrative analysis of tissue-specific gene regulatory variation with genome-wide association studies, we detected relevant tissue types and several candidate genes for a number of agronomic traits, including leaf during the day for the anthesis-silking interval, leaf during the day for kernel Zeinoxanthin level, and root for ear height, highlighting the potential contribution of tissue-specific gene expression to variation in agronomic traits. Using transcriptome-wide association and colocalization analysis, we associated tissue-specific expression variation of 74 genes to agronomic traits variation. Our findings provide novel insights into the genetic and biological mechanisms underlying maize complex traits, and the multi-tissue regulatory atlas serves as a primary source for biological interpretation, functional validation, and genomic improvement of maize.

## Teaser

This study will interest plant scientists and crop geneticists seeking to understand tissue-specific gene regulation in maize. Using 1,771 RNA-seq datasets from seven tissues, Lei et al. constructed a multi-tissue gene regulation atlas, revealing that 59% of eQTLs are tissue-specific. The authors linked 74 genes to 30 agronomic traits, highlighting tissue-driven trait variation. Their findings enhance fine-mapping and functional annotation in plant genetics, providing valuable insights for improving complex trait studies in crops.

## Introduction

Genome-wide association studies (GWAS) have identified numerous quantitative trait loci (QTLs) for crop agronomic traits (Lipka *et al*. 2013; Owens *et al*. 2014; Liu *et al*. 2020, 2021; Wang *et al*. 2020; Li *et al*. 2022; Jin *et al*. 2023). However, fine-mapping genes underlying those associations and dissecting the underlying biological mechanisms remain a significant challenge, partially due to a lack of knowledge of whether these variants act at the transcriptional or post-transcriptional level or in the functional tissue. Recently, a few studies have demonstrated the power of combining GWAS, transcriptome-wide association studies (TWAS) and expression quantitative trait loci (eQTL) analysis in functionally characterizing the association signals (Pang *et al*. 2019; Zheng *et al*. 2020; Lin *et al*. 2022). By leveraging population-scale transcriptome variation, many genes (Lin *et al*. 2017; Kremling *et al*. 2019) and co-expression networks (Schaefer *et al*. 2018) were revealed. Further integrative analyses of large-scale GWAS and multi-tissue regulatory datasets in the human Genotype-Tissue Expression (GTEx) project (GTEx Consortium 2020) and in the farm animal Genotype-Tissue Expression project (FarmGTEx) (Liu *et al*. 2022; Teng *et al*. 2024) have revealed the potential to discover tissue-specific regulatory variants (eQTLs) and connected trait-relevant tissues or cell types to complex trait variation, paving a promising way to obtain a comprehensive understanding on the genetic regulation of complex trait.

Maize (*Zea mays*), recognized for its extensive genetic diversity, is an important model species in genetic research. It is the most extensively cultivated crop worldwide and plays a vital role in sustaining the increasing global population. Although, transcriptome-wide association analysis in Rice revealed an important contribution from gene expression to panicle traits (Ming *et al*. 2023), no publication has comprehensively reported the causal tissue or tissue-specific regulatory variants relevant to agronomic trait variation in maize. Such a multi-tissue regulatory roadmap will offer valuable information for fine-mapping causal variants/genes underlying agronomic traits (Fang *et al*. 2020), functional characterization of the biological mechanisms (Yan *et al*. 2020; Pan *et al*. 2021; Zhao *et al*. 2021), and genetic improvement programs (Clark *et al*. 2020) in crop species.

Here, we reanalyzed a multi-tissue transcriptome dataset that included 1,771 RNA-seq samples from 7 tissues in a maize diversity panel with 298 inbred maize lines (Kremling *et al*. 2018). We constructed a multi-tissue atlas of regulatory variants, quantified the contribution of tissue-specific expression to agronomic trait variation, and connected the tissue-specific expression of 74 genes to the variation in 30 agronomic traits. The results presented here, for the first time, potentially established connections at the RNA level between tissues and agronomic traits in crops and provide an important starting point for post-GWAS functional experiments to explore genotype-phenotype relationships in crops.

## Materials and methods

### Data

298 inbred lines from the maize diversity panel were re-sequenced and called genotypes were downloaded from Kremling *et al*. (2018). 4,260,521 variants with minor allele frequency >0.05 and missing rate <0.99 were retained for downstream analysis. We downloaded the expression matrix for 7 tissues from Kremling *et al*. (2018). Genes expressed (normalized counts > 0) in more than 50% of the individuals were kept for downstream analysis. To account for hidden batch effects and other technical/biological sources of transcriptome-wide variation in gene expression, we estimated latent covariates in each tissue using the Probabilistic Estimation of Expression Residuals (PEER) method (Stegle *et al*. 2010). In each tissue, we used 25 PEER covariates to account for the effects of confounding variables on gene expression in following QTL analyses. To compare the effect sizes of detected eQTLs across tissues, we standardized the expression values to have mean 0 and variance 1. The best linear unbiased predictor (BLUP) values (Supplementary Table 11) for 30 kernel carotenoid traits, 20 kernel tocochromanol traits and 11 agronomic traits were downloaded from Owens *et al*. (2014), Lipka *et al*. (2013), and Peiffer *et al*. (2014), respectively. Besides the 11 agronomic traits, a phenotype file (traitMatrix_maize282NAM_v15-130212.txt) containing other 32 unique types of phenotypes scored in more than 1 environment, was downloaded from Panzea (Zhao *et al*. 2006). BLUP value for each inbred line was calculated across all environments using the mixed linear model in the R package “lme4” (Bates *et al*. 2015).

### Population structure analysis and expression data clustering analysis

We performed principal component analysis (PCA) with an identity-by-state (IBS) matrix estimated using 4,260,521 single-nucleotide polymorphisms (SNPs) and selected the first 2 principal components (PCs) to visualize the population structure using the *prcomp* function in R. We visualized sample clusters based on 15,505 genes detected in 7 tissues using Uniform Manifold Approximation and Projection (UMAP) approaches in the R package “uwot” (Melville *et al*. 2020).

### Detecting tissue-specific gene expression, function enrichment, and evolutionary analysis

We followed the approach presented in Finucane *et al.* (2018) to infer tissue-specific expressed genes. First, we scaled the log2-transformed expression (i.e. log2count) of genes to have a mean of 0 and a variance of 1. Then, we fitted the following model to calculate a *t*-statistic for each gene, 1 tissue at a time.


(1) y=μ+Xb+e

where *y* is an *n* × 7 vector of the scaled log2count with *n* being the sample size and 7 being the number of tissues. *μ* is the intercept, *X* is a dummy variable for tissue, where samples from the tested tissue (e.g. RT) were denoted as “1”, and samples from remaining tissues (e.g. SH, LB, LT, LD, and LN) were denoted as “−1”, *b* is the corresponding tissue effect, and *e* is the residual effect. We ranked genes in each tissue according to their *t*-statistic of *b* and chose the top 5% as candidate genes. Meanwhile, we filtered these genes by requiring a median fold change of 2 in relation to all the other tissues.

The “ClusterProfiler” R package (Yu *et al*. 2012) was used to perform functional enrichment, Gene Ontology (GO) and Kyoto Encyclopedia of Genes and Genomes (KEGG) analysis. We obtained the genome of teosinte TIL01 (*Zea mays* ssp. *Parviglumis*) from maizeGDB (https://download.maizegdb.org/Zv-TIL01-REFERENCE-PanAnd-1.0/) (Lawrence *et al*. 2004). The nonsynonymous (*d_N_*) and synonymous (*d_S_*) substitutions were computed for all orthologous genes between 2 entire genomes using the orthologr package (Drost *et al*. 2015).

### Genome-wide association analysis

GWAS was performed using the mixed linear model implemented in the *mlma* module from *GCTA* (Yang *et al*. 2012), adjusting for population structure with a genomic kinship matrix. We performed a conditional analysis where all top-associated SNPs (the SNPs with the highest *P*-value from each association QTL from the initial GWA scan) were included as covariates for additional association signals. We performed this conditional analysis repeatedly until there were no more SNPs that surpassed the significance threshold. This conditional analysis was performed using the *cojo* module (Yang *et al*. 2012) from *GCTA* (Yang *et al*. 2011).

In this study, Bonferroni correction was used to derive the significance threshold. To avoid being over conservative, we estimated the effective number of independent markers (Me) using the GEC software (Li *et al*. 2012) and derived a less conservative genome-wide significance threshold following 0.05/Me (2.02× 10^−8^ equivalent to −Log_10_  *P* = 7.69).

### Estimating the contribution from transcriptome variation to agronomic trait variation and transcriptome-wide association analysis

A linear mixed model was used to estimate the amount of phenotypic variance explained (PVE) by transcriptome variation.


(2) y=μ+Wu+e

where *y* is an *n* × 1 vector of phenotypic values with *n* being the sample size, *u* is an *m* × 1 vector of the joint effects of all genes on the phenotype, and *e* is an *n* × 1 vector of residuals. *W* is the corresponding design matrix obtained from a Cholesky decomposition of the transcriptomic relationship matrix *A*, estimated from the transcriptome with OSCA (Zhang *et al*. 2019). The *W* satisfied $A=WW^{T}$; therefore, u and e are random effect with $u∼N\;(0,\;I\sigma_{u}^{2})$ and $\;e∼N\;(0,\;I\sigma_{e}^{2})$. The proportion of variance explained by transcriptome variation was the intraclass correlation $\rho^{2}=\sigma_{u}^{2}/(\sigma_{u}^{2}+\sigma_{e}^{2})$. TWAS was computed by mixed-linear model approaches using OSCA (Zhang *et al*. 2019). Bonferroni-corrected thresholds (0.05/genes number, 2.58× 10^−6^) and (0.05/associated genes number, 1.11× 10^−3^) were used as the significance threshold and lenient significance threshold, respectively.

### Colocalization of eQTLs and QTLs

We downloaded previously reported maize QTLs from a number of published studies (Lipka *et al*. 2013; Owens *et al*. 2014; Liu *et al*. 2020, 2021; Wang *et al*. 2020; Li *et al*. 2022; Jin *et al*. 2023). A list of these QTLs is summarized in Supplementary Table 12. To adjust for differences in reference genomes, we lifted over both eQTLs and QTLs with reported position from maize genome AGP v3 to AGP v5 using online tools from EnsemblPlants (https://plants.ensembl.org/Zea_mays/Tools/AssemblyConverter?db=core, Supplementary Table 6).

To compile a list of eQTLs associated with multi-tissue gene expression, we grouped eQTLs from multiple tissues with linkage disequilibrium > 0.2 and physical distance < 500 kb as a single eQTL. Since linkage disequilibrium structure varies between populations in which the trait QTLs were detected, trait QTLs and eQTLs were regarded as colocalized when their physical distance was <100 bp. We used summary-based Mendelian randomization (SMR) (Zhu *et al*. 2016) to infer the effect of gene expression variation on traits using eQTL.

### Validation of gene-trait correlations

RNA sequencing reads of the Complete-diallel design plus Unbalanced Breeding-like Inter-Cross (CUBIC) population were downloaded on NCBI with Bioproject accession number PRJNA597703 (Liu *et al*. 2020). Trimmomatic (Bolger *et al*. 2014) version 0.32 was used to remove the adaptor and the low-quality reads. STAR aligner (Dobin *et al*. 2013) v.2.4.2a was used to align reads against the maize genome annotation version AGPv.3.29. FeatureCounts (Liao *et al*. 2014) was used to obtain gene-level counts from the resulting BAM files. Counts were normalized by library sequencing depth using the -estimateSizeFactors method in DESeq2 (Love *et al*. 2014) in R.

## Results

### Data summary

**Fig. 1.**
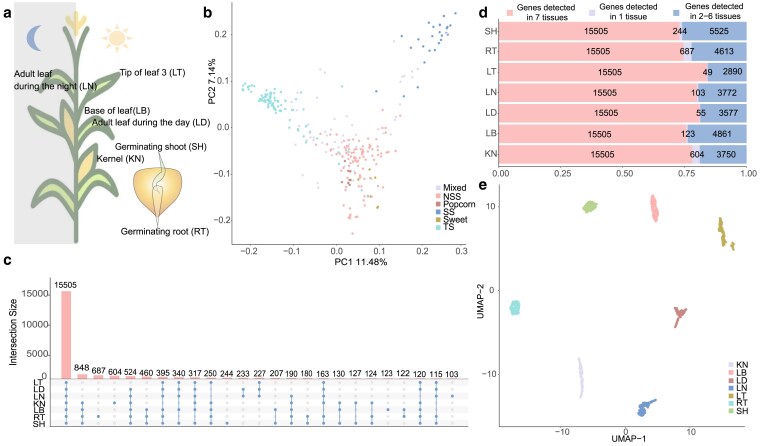
Gene expression diversity across 7 tissues in the maize diversity panel. a) Graphical illustration of the 7 tissues analyzed in our study. b) PCA of the IBS matrix estimated from 4,260,521 SNPs. c) Overlap of genes among 7 tissues. d) The number of genes detected in 7 tissues, genes detected in 1 tissue, and genes detected in 2 to 6 tissues. e) UMAP projection of 15,505 genes detected in all tissues.

We downloaded 1,960 publicly available RNA-seq datasets from a maize diversity panel with 298 inbred lines. These lines included 26 stiff stalk lines, 103 nonstiff stalk lines, 77 tropical/subtropical lines, 6 sweet corn lines, 9 popcorn lines, and 61 mixed lines (Supplementary Table 1). After quality control, 1,771 datasets from 7 tissues were kept for downstream analysis: germinating root (RT), germinating shoot (SH), base of leaf 3 (LB), tip of leaf 3 (LT), kernel (KN), adult leaf during the day (LD), and adult leaf during the night (LN, Fig. 1a) were kept for downstream analysis. Meanwhile, we downloaded 4,263,832 SNPs from Kremling *et al.* (2018) and kept 4,260,521 SNPs after quality control (Materials and methods).

### Multi-tissue gene expression diversity in a maize diversity panel

The first PC of the IBS matrix, estimated from genome-wide SNPs, explained 11.48% (Fig. 1b) of the overall variation, indicating the presence of moderate population structure. We detected an average of 19,913 genes (median = 19,859, ranging from 18,444 to 21,274 with normalized counts > 0 in more than 50% lines) across 7 tissues, amounting to 24,399 genes with detectable expression in 7 tissues. To explore the multi-tissue gene expression diversity, we first performed pairwise comparisons for the genes expressed across all 7 tissues. Overall, 15,505 genes (64%) were detected in all tissues (Fig. 1c), while 1.31% of genes (range from 49 to 687, median = 123) were uniquely detected in 1 tissue (Fig. 1d) on an average level. When projecting the expression matrix of 15,505 genes detected in all 7 tissues using UMAP (Fig. 1e), gene expression separated by tissue type. These results indicated that tissue specificity rather than genetic distance dominated expression variation in this dataset.

### Detection and functional characterization of the tissue–specific genes

**Fig. 2.**
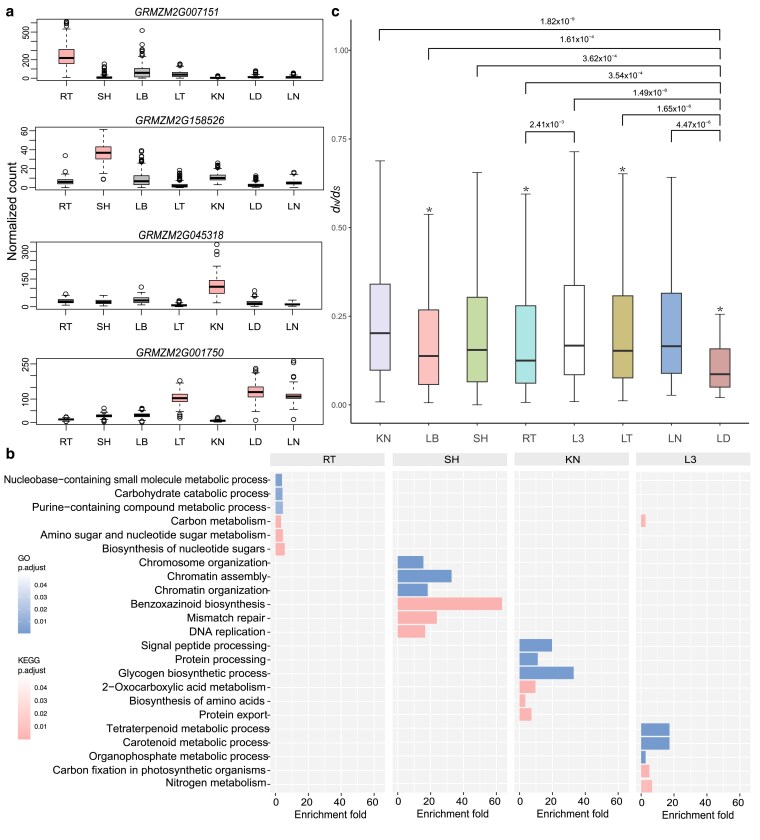
Characteristics of the tissue-specific gene expression in maize. a) Examples of selected tissue-specific genes in RT (*GRMZM2G007151*), SH (*GRMZM2G158526*), KN (*GRMZM2G045318*), and leaf, including LT, LD, and LN (*GRMZM2G001750*). *y*-axis is the gene expression. b) GO and KEGG enrichment analysis of tissue-specific genes (the top 5% of genes based on *t*-statistics and median of expression). The value for each bar is the fold of enrichment. c) *d_N_/d_S_* ratio between maize and teosinte for tissue-specific genes across 7 tissues (L3 stands for LT, LN, and LD). *P*-values were calculated using Student's *t*-test. For each tissue, we compared tissue-specific genes of this tissue against the remaining genes using a 2-tailed *t*-test; “*” indicates *P* < 0.01.

We detected tissue-specific expressed genes 1 tissue at a time based on the rank of *t*-statistics and expression fold change (Materials and methods, Supplementary Table 2). For each studied tissue, there were 37 to 317 (median = 185) genes specifically expressed. The number of tissue-specific expressed genes obtained at population scale was significantly lower than that obtained from a single reference accession B73 (Supplementary Fig. 1), suggesting that tissue-specific expression varies considerably among different genetic background. There were a number of genes, with known biological functions that were highly expressed in a particular tissue across all the inbred lines, indicating their potential functional relevance in the corresponding tissue (Fig. 2a). For example, *GRMZM2G045318*, a transport protein Sec61 beta subunit, which is involved in the translocation of secretory proteins into the endoplasmic reticulum (Zhao and Jäntti 2009), was specifically expressed in kernel. *GRMZM2G001750*, which encodes an alpha/beta-hydrolases superfamily protein that has been proven to function as bona fide hormone receptors in the strigolactone (a novel carotenoid-derived plant hormone), and gibberellin response pathways (Mindrebo *et al*. 2016), was specifically expressed in leaf (Fig. 2a). We also found that *GRMZM2G007151* is specifically expressed in RT, and *GRMZM2G158526* is specifically expressed in SH (Fig. 2a), which requires further experimental investigation.

Overall, the number of tissue-specific expressed genes were functionally enriched in tissue-relevant pathways or biological processes (Fig. 2b, Supplementary Fig. 2, Supplementary Table 3). For example, KN-specific genes were enriched for glycogen biosynthesis (FDR adjusted *P*-value = 7.31× 10^−4^, enrichment fold = 32.94, Fig. 2b), and signal peptide processing. (FDR adjusted *P*-value = 2.06× 10^−4^, enrichment fold = 19.76, Fig. 2b). Leaf-specific genes were significantly enriched for carbon fixation in photosynthetic organisms (FDR adjusted *P*-value = 8.21× 10^−4^, enrichment fold = 4.98 Fig. 2b), and nitrogen metabolism (FDR adjusted *P*-value = 6.22× 10^−3^, enrichment fold = 6.64, Fig. 2b).

To explore the evolutionary conservation of tissue-specific genes, we explored *d_N_/d_S_* ratios of orthologous genes between maize and teosinte in 7 tissues. LD-specific genes displayed the lowest *d_N_/d_S_* ratios (median = 0.16), while KN-specific genes had the highest *d_N_/d_S_* ratios (median = 0.20, Fig. 2c). It is likely that, in constrained tissues (e.g. LD, not under strong and directional selection), tissue-specific genes tend to evolve slowly, whereas, in the relaxed tissues (e.g. KN, under strong and directional selection), tissue-specific genes evolved more rapidly, revealing the importance of tissue-driven evolution.

### Genetic basis of multi-tissue gene expression diversity

**Fig. 3.**
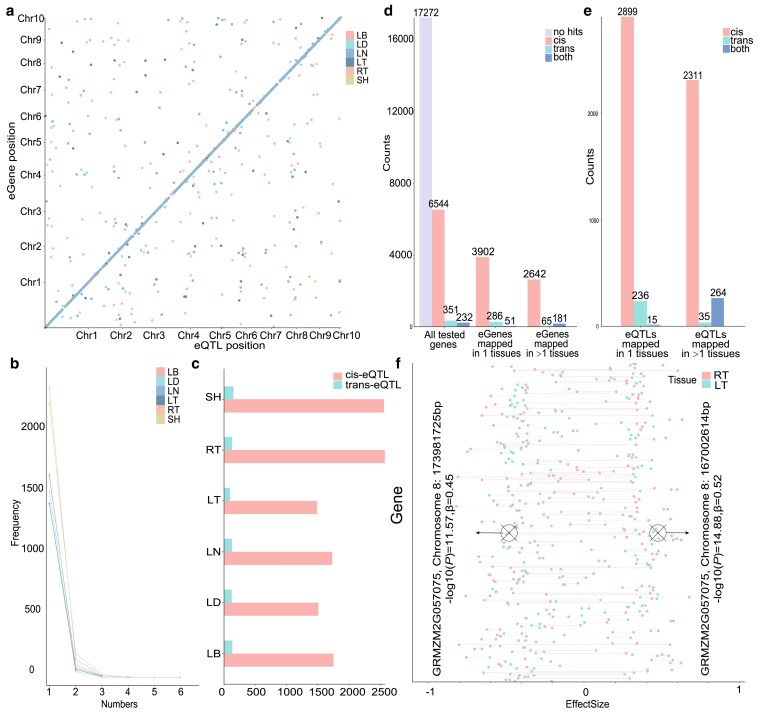
Summary of the eGWAS results. a) A multi-tissue atlas of maize regulatory variants constructed from 6 tissues (kernel was excluded due to a significantly lower number of detected eQTLs). *x*-axis is the location of the eQTL, while the *y*-axis is the position of the associated gene. b) The number of eQTLs associated with 1 gene in each of the 6 tissues. c) The number of cis and trans eQTLs identified in each tissue. d) Tissue specificity of eGenes. e) The number of eQTLs mapped in 1 and more tissues. f) Estimated eQTL effect sizes (*x*-axis) in RT and LT for all the eGenes (*y*-axis). Pink lines represent the eQTL have opposite effect in 2 tissues. Triangle symbols represent the eQTL for *GRMZM2G057075*.

To explore the genetic basis of multi-tissue gene expression diversity, we performed a comprehensive catalogue of the genetic variants underlying expression diversity in 7 tissues and built a multi-tissue atlas of regulatory variants. In total, we detected 5,760 eQTLs for 7,127 genes (eGenes) in 6 tissues after grouping eQTLs with linkage disequilibrium > 0.2 and physical distance < 500 kb as a single eQTL (Fig. 3a, kernel was excluded due to a significantly lower number of detected eQTLs, Supplementary Table 4). The number of eGenes varied considerably from 1 tissue to another, ranging from 1,506 in LT to 2,543 in RT (median = 1,762.5). The majority of eGenes had 1 eQTL, while very few eGenes associated with up to 6 eQTLs (Fig. 3b). Across all tissues, the number of cis-eQTLs (1 Mb of the target gene's transcription start site) was significantly larger than that for trans-eQTLs (Fig. 3c), which was consistent with previous eQTL mapping reports (Li *et al*. 2013).

To explore the similarities and differences in gene expression regulation across tissues, we compared the overlap and effect sizes of detected eQTLs. 59% (4239/7127) of eGenes showed eQTLs in only 1 tissue type (Fig. 3d), and 55% (3150/5760) of eQTL were detected in 1 tissue type (Fig. 3e), suggesting that gene expression regulation was generally tissue-specific. In addition, the sign and direction of eQTL effect sizes changed considerably across tissues. For example, between the 2 most distinctive tissues, RT and LT (Pearson's *r* = 0.09, Fig. 3f), 19.7% (45/228) of eQTLs showed effect sizes in opposite directions. Although, a single eQTL on Chromosome 8: 167,002,614 bp for *GRMZM2G057075* (homologous to *Arabidopsis thaliana* abiotic stress response repressor CLB; De Silva *et al*. 2011) was simultaneously detected in RT and LT. The G allele increased CLB expression in RT but decreased in LT.

### Multi-tissue transcriptome-wide association analysis highlights the relationship between tissue-specific gene expression and variation in agronomic traits

**Fig. 4.**
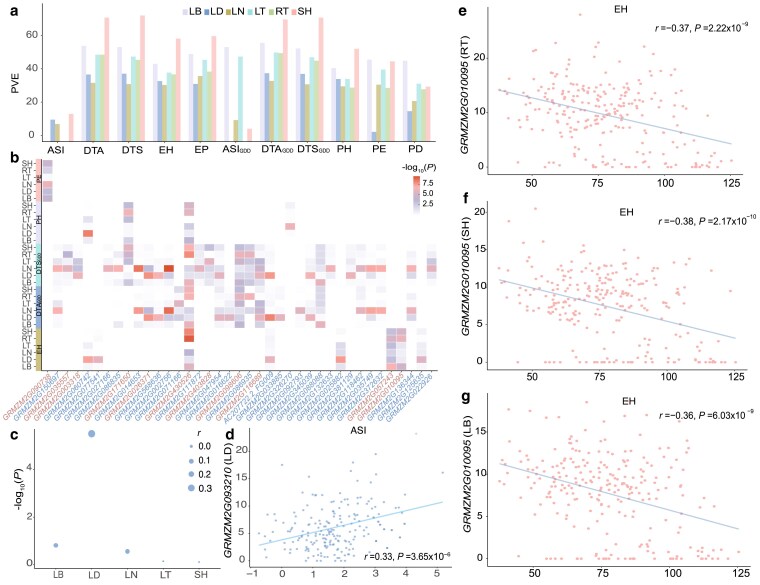
Contribution of tissue-specific gene expression to variation in agronomic traits. a) The proportion of PVE by gene expression in 7 tissues. b) A heatmap illustrating the *P*-values of TWAS. Each cell represents a *P*-value testing the association between the expression of a gene (*x*-axis) in specific tissue and a trait (*y*-axis). The red color highlights genes from category II, while blue represents genes belonging to category I. c, d) Expression-to-trait correlation and *P*-value testing the association between *GRMZM2G093210* expression in 5 tissue (RT excluded due to undetectable expression) and ASI variation. e–g) Expression-to-trait correlation maps for *GRMZM2G010095* and EH in RT, SH, and LB.

To estimate the contribution of multi-tissue transcriptome variation to agronomic trait variation, we calculated the proportion of PVE by the 24,399 genes using a linear mixed model, 1 tissue at a time (Fig. 4a, Materials and methods). In this analysis, 11 agronomic traits, measured on the same population (Peiffer *et al*. 2014), were analyzed, including growing degree-days to silking (DTS_GDD_), growing degree-days to anthesis (DTA_GDD_), growing degree-days to the anthesis-silking interval (ASI_GDD_), DTS, days to anthesis (DTA), the ASI, plant height (PH), ear height (EH), the distance between PH and EH (PE), the ratio between EH and PH (EP), and ratio between PH and DTA (PD) etc. (Supplementary Table 9). Overall, there was considerable variation in the amount of variance explained by each tissue (median from 36.74 to 61.55%, Fig. 4a). Taking DTS as an example, the SH transcriptome explained 75.45% of the phenotypic variation, while the LN transcriptome only explained 34.33%. Across 6 tissues (ranging from 29.73 to 52.88%), the SH transcriptome explained the highest phenotypic variance (median = 61.55% for the 11 agronomic traits), followed by the LB transcriptome (median = 53.99%). These results revealed a variable contribution from tissue-specific transcriptome diversity to agronomic trait variation.

To explore the relationship between tissue-specific gene expression and variation of agronomic traits, we performed multi-tissue transcriptome-wide association analysis 1 tissue at a time for 43 traits. In total, 87 genes were detected with significant association to at least 1 trait at genome-wide threshold (Fig. 4b, Supplementary Table 5). Consistent with previous reports, 4 out of the 87 genes were reported as important regulators for the corresponding trait (Li *et al*. 2019; Liang *et al*. 2019). There were drastic differences in the number of associated genes detected for different tissues. For instance, only 7 genes were identified in LT, while 28 genes were identified in LD. Based on TWAS *P*-values obtained from different tissues (Fig. 4b), we classified those genes into 2 categories. The first category included 60 (69%) genes with expression in only 1 tissue associated with agronomic trait variation even at a lenient significance threshold correcting for multiple testing of 87 genes (*P* = 0.05/87 = 5.75× 10^−4^), whereas the second category had 27 (31%) genes with expression in multiple tissues associated with agronomic trait variation. For example, *GRMZM2G093210*, a homolog of *A. thaliana* lectin-like receptor kinase 7 essential for pollen development (Wan *et al*. 2008), showed a positive correlation with ASI (TWAS *P* = 1.46× 10^−6^, Fig. 4c and d) in LD but not the remaining tissues studied here. In contrast, *GRMZM2G010095*, a homolog to *Gossypium hirsutum* C2 NT-type domain-containing protein (Wang *et al*. 2016), was associated with EH in RT (TWAS *P* = 8.54× 10^−7^), SH (*P* = 7.51× 10^−6^) and LB (*P* = 1.64× 10^−5^, Fig. 4e–g). Overall, these results highlighted the role of tissue-specific expression in the biological interpretation of genes associated with agronomic traits, with potential benefits in the functional characterization of the underlying mechanisms.

### Colocalization of eQTLs and QTLs associated with agronomic trait

**Fig. 5.**
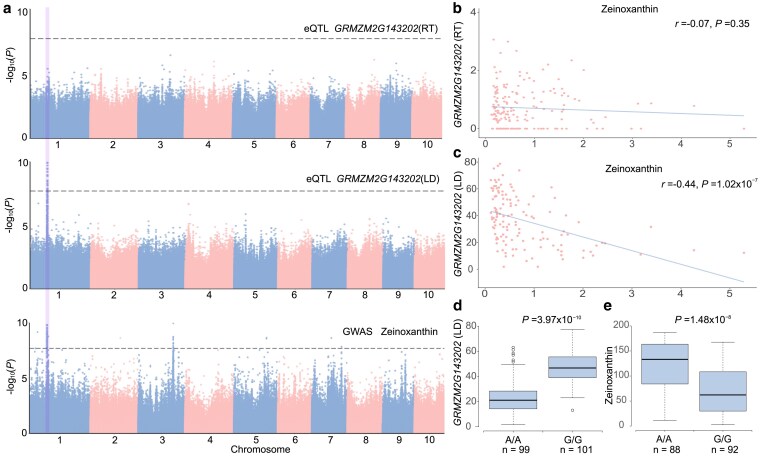
Colocalization of eQTL regulating *GRMZM2G143202* expression in RT and LD, and QTL associated with Zeinoxanthin on Chromosome 1: 87,808,526 bp. a) Manhattan plots for *GRMZM2G143202* eQTL mapping in RT, LD, and zeinoxanthin levels. The vertical line highlights the eQTL and QTL position on Chromosome 1: 87,808,526 bp. Gene expression-to-phenotype maps. *GRMZM2G143202* expression in LD b) was negatively correlated with Zeinoxanthin, whereas gene expression in RT c) was not. Boxplot of *GRMZM2G143202* expression d) and zeinoxanthin e) on Chromosome 1: 87,808,526 bp. *P*-values were obtained from GWAS.

To link QTLs associated with agronomic traits and eQTLs, we downloaded 2,941 previously reported QTLs associated with variation in 60 traits, including 50 agronomic traits and 10 metabolic traits (Lipka *et al*. 2013; Owens *et al*. 2014; Liu *et al*. 2020, 2021; Wang *et al*. 2020; Li *et al*. 2022; Jin *et al*. 2023) (Materials and methods, Supplementary Table 6). In total, 2,801 QTL comprising all traits have at least 1 eQTL colocolized within 500 kb, including 4,290 genes (Supplementary Table 7). Furthermore, 1% (33) agronomic trait QTLs were colocalized with at least 1 eQTL within 100 bp, including 16 genes and 14 agronomic traits (Materials and methods, Supplementary Table 8). To evaluate whether the eGenes or eQTLs was more likely to be associated with the variation in corresponding agronomic traits, we conducted 2 comparisons. The first comparison involved analyzing the TWAS *P*-values of eGenes against those derived from a set of randomly selected genes. We found significantly smaller *P*-values for colocalized eGenes (Supplementary Fig. 3, *P* = 4.55× 10^−7^), suggesting that they were more likely to be associated with agronomic trait variation. Additionally, we compared proportion of PVEs by eQTLs associated with these eGenes and PVE explained by randomly selected SNPs. For 63% (27/43) of the traits (Supplementary Fig. 4), eQTLs exhibited a significantly higher PVE than that from randomly selected SNPs. Among the colocalized eGenes, 12 genes were tissue-specific and associated with the variation of 11 agronomic traits, again emphasizing the importance of tissue-specific gene expression in agronomic trait variation. For example, a QTL on Chromosome 1: 87,808,526 bp was associated with both zeinoxanthin and *GRMZM2G143202* expression in LD (Fig. 5a–c), which potentially have a causal relationship (*P*_SMR_ = 1.73× 10^−5^, *P*_heidi_ = 0.88). The G allele increased the expression of *GRMZM2G143202* and decreased zeinoxanthin levels (Fig. 5d and e).

To validate the correlation between genes and traits, we downloaded a second dataset with transcriptome from leaf and 14 phenotypes from 500 inbred lines (Materials and methods). Although this population differed in ancestry and harbored a different set of genetic variants, 39% (36/92) of the gene-traits associations were replicated (*P* < 0.05, Supplementary Table 9 and Fig. 5).

## Discussion

Consistent with previous reports from the Human and Farm GTEx projects (GTEx Consortium 2020; Liu *et al*. 2022; Teng *et al*. 2024), we revealed large variation in the proportion of agronomic trait variance explained by transcriptome differences across tissues (Fig. 4a) and detected genes with tissue-specific contribution to agronomic traits (Fig. 4b).

One of the motivations to initialize this GTEx project was to detect tissue-specific regulatory variants (eQTLs) and connect trait-relevant tissues or cell types to complex trait variation. The Human and Farm GTEx projects typically involved more than 50 tissues on hundreds of individuals (GTEx Consortium 2020; Liu *et al*. 2022; Teng *et al*. 2024). In absence of such a population scale dataset with multi-tissues, our study only represents a pilot study in plant. Although our study may be underpowered in terms of limited tissue types and small population size, we detected candidate tissue-specific genes with reported biological functions to relevant traits (*GRMZM2G045318* and *GRMZM2G001750* presented above) and found 9 candidate genes with potential tissue-specific contribution to agronomic trait variation though literature review (Supplementary Table 10). For example, *Rap2.7* (*GRMZM2G700665*) encode an AP2 transcription factor, involved in maize brace roots development (Li *et al*. 2019; Liang *et al*. 2019). Our analysis revealed a significant association between the expression of *Rap2.7* in RT and EH, and a RT-specific eQTL on Chromosome 8:131,596,242 bp regulating the expression of *Rap2.7*, suggesting a unique link among the eQTL, *Rap2.7* expression in RT and EH variation. These findings are valuable for interpreting previous GWAS results and justify a comprehensive functional characterization of the detected genes, tissue and trait connection. In addition, our analysis revealed 33 genes associated with more than 1 trait in a tissue specific manner. For example, *AC207722.2_FG009,* annotated as a chlorophyll a-b binding protein, was significantly associated with DTA_GDD_, DTS_GDD_ and, EH in LD (Supplementary Fig. 6). Although we revealed statistically significant association between tissue specific expression to complex trait variation, an important limitation of our study is that it lacks experimental validation. Therefore, further investigations are warranted to validate this association and if validated, elucidate the underlying mechanisms by which these genes exert their pleiotropic effects and to uncover their broader functional significance in the context of the studied traits.

It is important to note that another limitation of our study is the lack of a comprehensive genotype-to-phenotype map for maize agronomic traits. Our attempt to assemble such a map was challenged by a lack of publicity in releasing phenotypic data, discordance in genome assembly, and differences in marker density. These limited the power of leveraging a multi-tissue gene expression regulatory variant atlas to dissect the genetic basis of agronomic traits in maize. Nevertheless, with an increased amount of public data being available, such as CUBIC population (Liu *et al*. 2020) and 1,604 utilized maize inbred lines (Li *et al*. 2022), the findings presented in our study will make a larger contribution to the genetic dissection of maize complex traits. Another potential for the gene expression regulatory variant atlas is enhancing genomic prediction. Integrating tissue-specific genes into extended prediction models in cattle exhibited a modest increase in prediction accuracy compared with traditional models (Fang *et al*. 2017).

In summary, we present a multi-tissue maize gene expression regulatory variant atlas by analyzing 1,771 RNA-seq samples from 7 tissues. It represents an extensive reference resource for a detailed characterization of the genetic regulation of gene expression across multiple maize tissues. By establishing links between SNPs, tissue-specific genes expression, and agronomic trait variation, we highlighted a variable contribution from tissue-specific expression to agronomic variation and connected relevant tissues/genes for agronomic traits. The presented results demonstrate the feasibility of uncovering genetic regulatory variants in the maize transcriptome and connecting tissue-specific gene expression to agronomic traits by leveraging public data.

## Supplementary Material

jkaf140_Supplementary_Data

## Data Availability

The genotypes were downloaded from https://datacommons.cyverse.org/browse/iplant/home/shared/commons_repo/curated/Qi_Sun_Zea_mays_haplotype_map_2018/hmp321_unimputed. The processed expression counts were downloaded from https://de.cyverse.org/data/ds/iplant/home/shared/panzea/dataFromPubs. Kernel carotenoid BLUPs were obtained from Supplementary Table 1 of the study (https://doi.org/10.1534/genetics.114.169979), available at: https://pmc.ncbi.nlm.nih.gov/articles/instance/4256781/bin/supp_114.169979_Table S1.xlsx. Kernel tocochromanol BLUPs were downloaded from Table S1 of the study (https://doi.org/10.1534/g3.113.006148), available at: https://pmc.ncbi.nlm.nih.gov/articles/instance/3737168/bin/supp_g3.113.006148_Table S1.pdf. The agronomic trait BLUPs were downloaded from https://datacommons.cyverse.org/browse/iplant/home/shared/panzea/phenotypes/Peiffer2014Genetics_blupPhenos20150325.xlsx and https://datacommons.cyverse.org/browse/iplant/home/shared/panzea/phenotypes/traitMatrix_maize282NAM_v15-130212.txt. All data from this manuscript is publicly available in previous publications referenced in the Materials and methods. All codes used for analysis are available in the GitHub repository https://github.com/leilei37/maizeGTEx. Supplemental material available at G3 online.
