# Supplementary material for: Comprehensive analysis of 1,771 transcriptomes from 7 tissues enhance genetic and biological interpretations of maize complex traits
Source: G3 (Bethesda). 2025 Jul 22;15(9):jkaf140. doi: 10.1093/g3journal/jkaf140 (PMC12405892; doi:10.1093/g3journal/jkaf140)
Supplement: jkaf140_Supplementary_Data [file jkaf140_supplementary_data.zip › Supplemental_Figures_G3-2025-405809.docx]

Supporting information for“**Comprehensive analysis of 1771 transcriptomes from seven tissues enhance genetic and biological interpretations of maize complex traits ”**


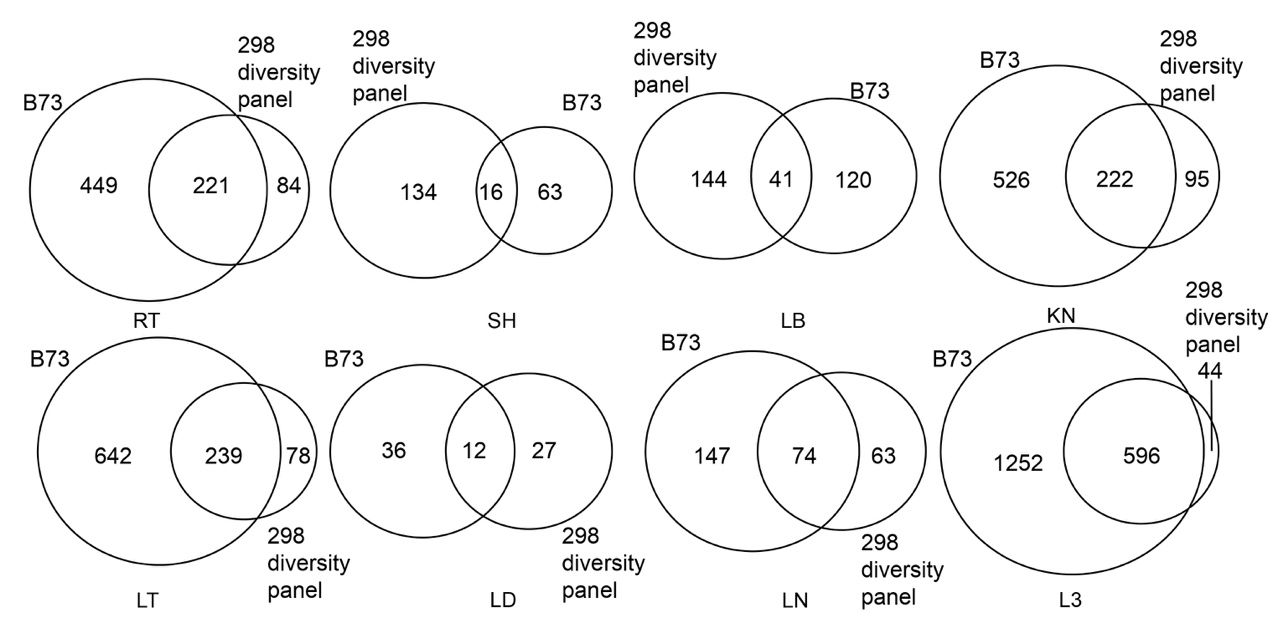



**Supplementary Fig S1. Venn diagram illustrating overlap of tissue-specific genes from B73 and the maize diversity panel across seven tissues.** L3 stands for LT, LN, and LD


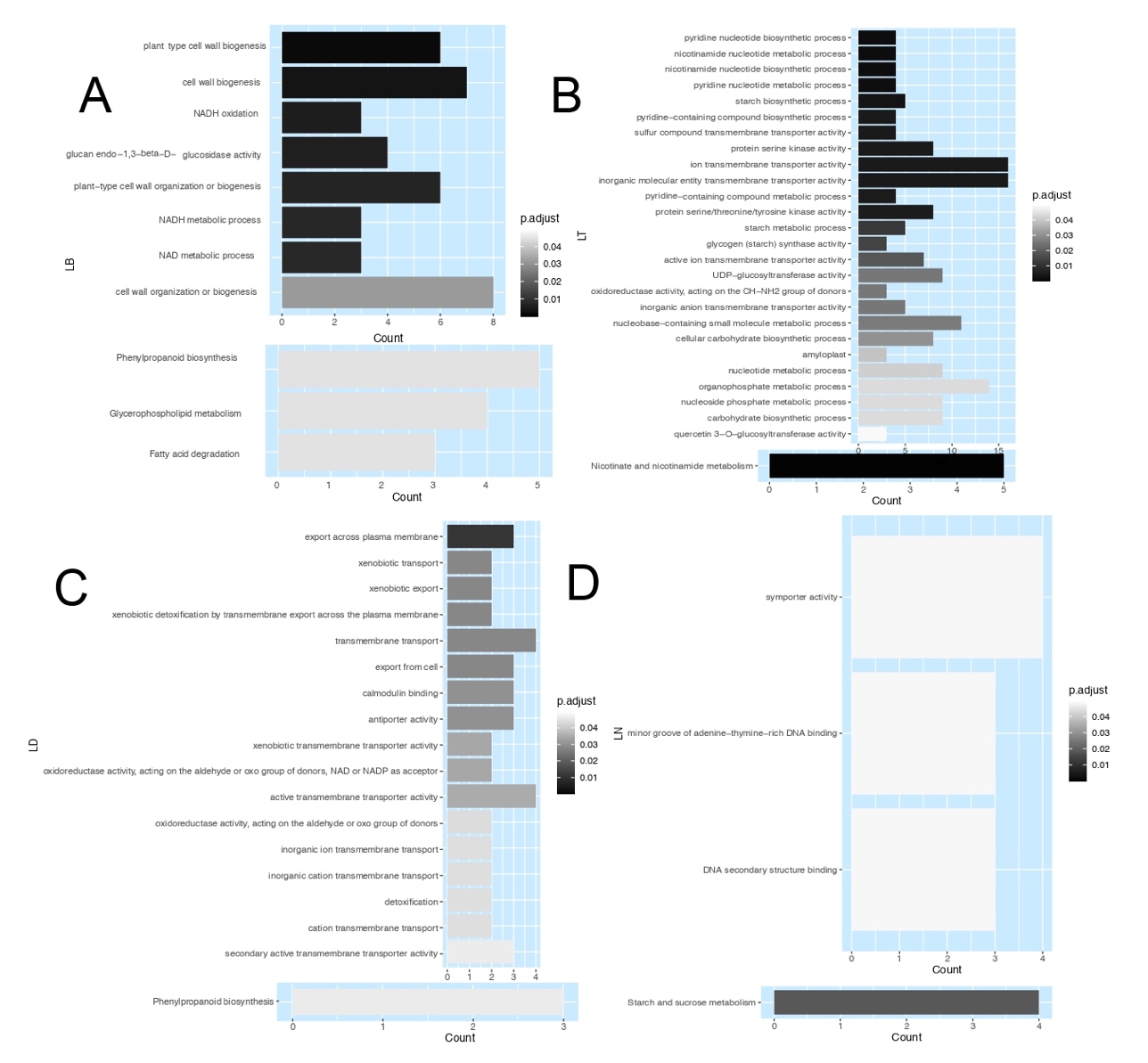


**Supplementary Fig S2. Gene Ontology (GO) and Kyoto Encyclopedia of Genes and Genomes (KEGG) enrichment analysis of tissue specific genes from LB(A), LT(B), LD(C), and LN(D).** The color corresponds to the -Log10 (adjusted *P*-value).


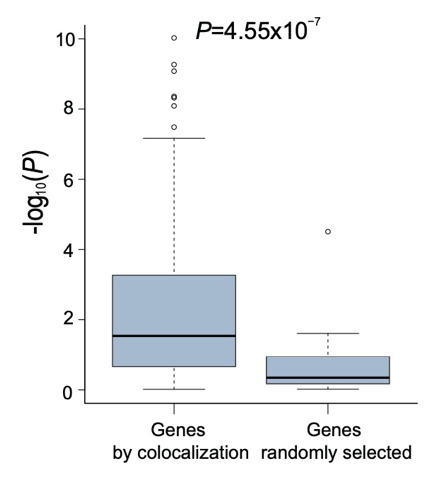


**Supplementary Fig S3. Comparison of TWAS *P*-values for genes whose eQTL was colocalized with agronomic traits QTLs and randomly selected genes.** *P*-values were calculated using Student’s *t*-test.


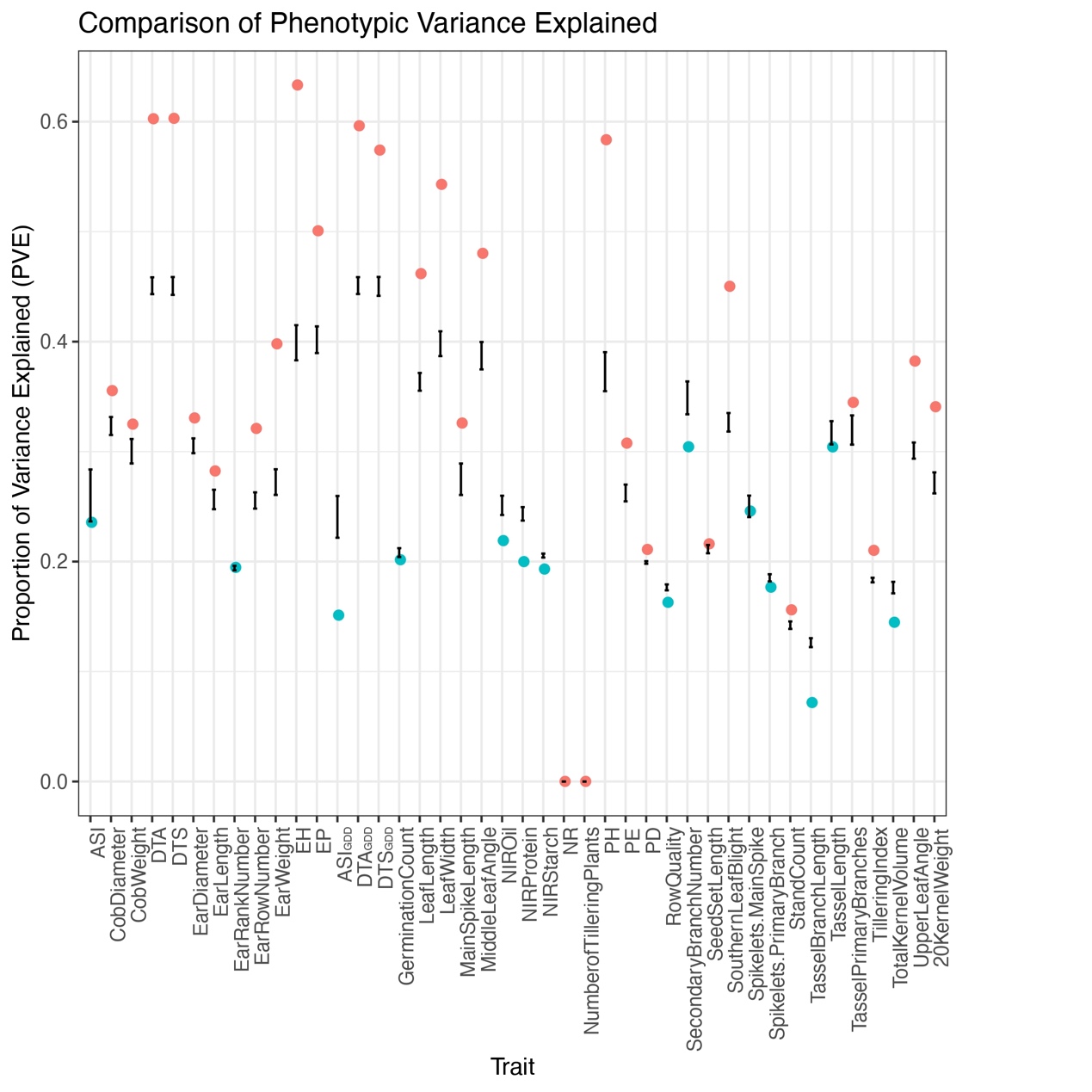


**Supplementary Fig S4. Comparison of proportion of phenotypic variance explained by eQTL associated with these eGenes and PVE explained by randomly selected SNPs.** Pink spots represent the PVE attributed to eQTLs associated with these eGenes, which exceeds the PVE of randomly selected SNPs by more than 100 times, as indicated by the 95% confidence interval (CI).

**
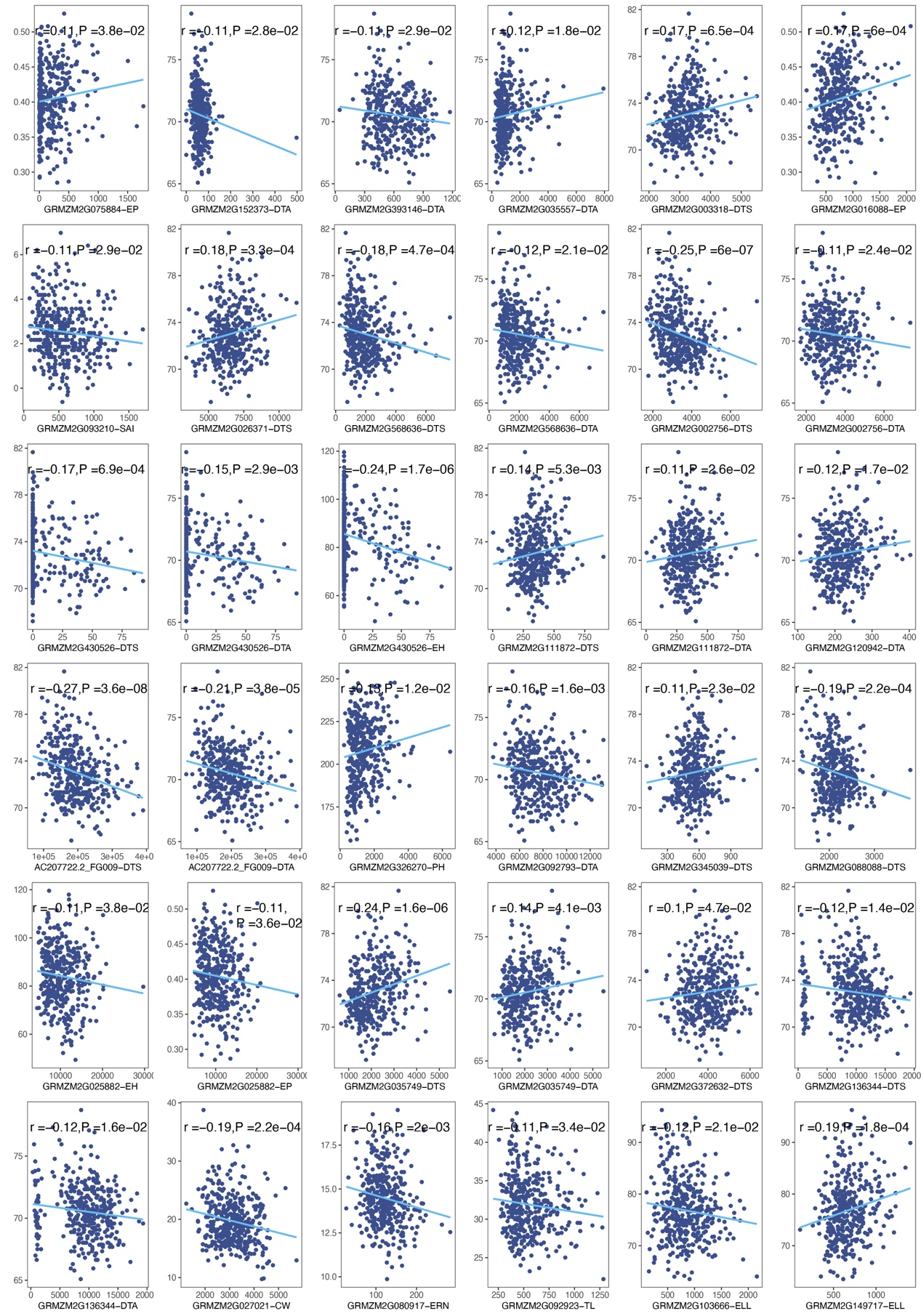
**

**Supplementary Fig S5. Scatter plot illustrating the relationship between gene expression and trait in CUBIC population.**


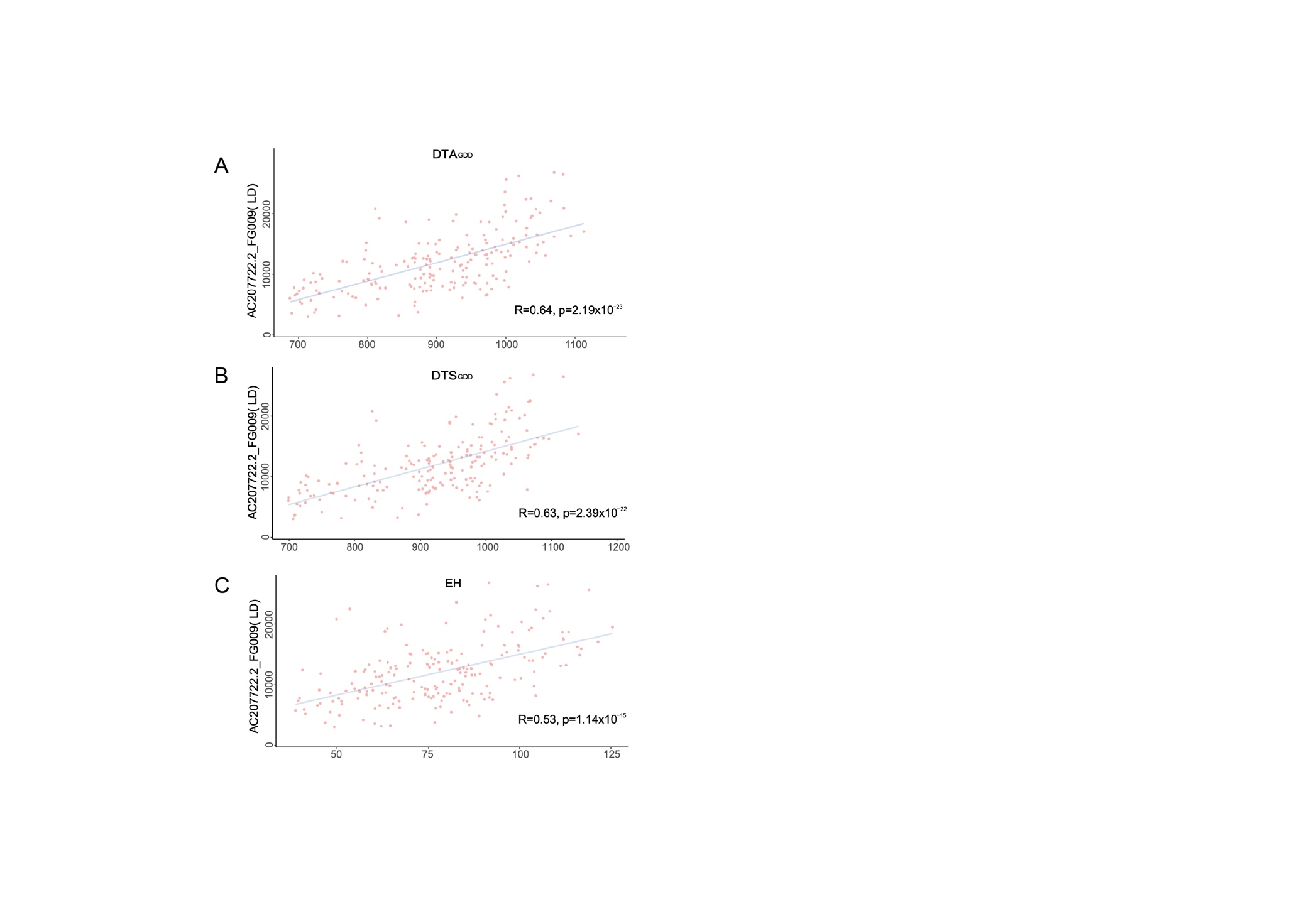


**Supplementary Fig S6.** **Scatter plot illustrating the relationship between *AC207722.2_FG009* expression and, DTA_GDD_(A), DTS_GDD_(B) and, EH(C) in LD.**
